# Comparison of the occlusal contact area of virtual models and actual models: a comparative in vitro study on Class I and Class II malocclusion models

**DOI:** 10.1186/s12903-018-0566-7

**Published:** 2018-06-19

**Authors:** Hyemin Lee, Jooly Cha, Youn-Sic Chun, Minji Kim

**Affiliations:** 1 Graduate School of Clinical Dentistry, Ewha Womans University, Seoul, South Korea; 2 Department of Orthodontics, College of Medicine, Ewha Womans University, Seoul, South Korea

**Keywords:** Digital intraoral scanner, Occlusal contact areas, Virtual occlusion

## Abstract

**Backgrounds:**

The occlusal registration of virtual models taken by intraoral scanners sometimes shows patterns which seem much different from the patients’ occlusion. Therefore, this study aims to evaluate the accuracy of virtual occlusion by comparing virtual occlusal contact area with actual occlusal contact area using a plaster model in vitro.

**Methods:**

Plaster dental models, 24 sets of Class I models and 20 sets of Class II models, were divided into a Molar, Premolar, and Anterior group. The occlusal contact areas calculated by the Prescale method and the virtual occlusion by scanning method were compared, and the ratio of the molar and incisor area were compared in order to find any particular tendencies.

**Results:**

There was no significant difference between the Prescale results and the scanner results in both the molar and premolar groups (*p* = 0.083 and 0.053, respectively). On the other hand, there was a significant difference between the Prescale and the scanner results in the anterior group with the scanner results presenting overestimation of the occlusal contact points (*p* < 0.05). In Molars group, the regression analysis shows that the two variables express linear correlation and has a linear equation with a slope of 0.917. R^2^ is 0.930. Groups of Premolars and Anteriors had a week linear relationship and greater dispersion.

**Conclusions:**

Difference between the actual and virtual occlusion revealed in the anterior portion, where overestimation was observed in the virtual model obtained from the scanning method. Nevertheless, molar and premolar areas showed relatively accurate occlusal contact area in the virtual model.

## Background

The development of digital scanners brings about a lot of changes to the dental treatment environment. As a non-invasive method without radiation exposure, the digital scanning method is expected to reduce treatment time and improve the quality of treatment by allowing frequent evaluations according to needs [1]. It is even possible to know the state of occlusal contact by utilizing digital imagery by scanning models using an intraoral scanner. The use of the intraoral scanner offers the advantage of excluding the necessity of the impression process, production of the master cast, errors in the process of mounting on the articulator and errors due to the thickness of the occlusal evaluating paper. The manners of recording the interocclusal relationship include scanning the impression material of the maxillo-mandibular occlusal relationship, and scanning the buccal portion at centric occlusion. There is a risk of movement of the impression material during the scanning process in the former method, so the latter method which offers useful information in the analysis of the state of occlusion in an actual patient without the need of any impression materials by directly capturing the occlusion and buccal contact points is preferred.

Digital impressions are divided into a direct method and an indirect method. The direct method is where digital imagery is obtained by scanning the oral environment of the patient, and the indirect method is where images in the form of digital models are obtained by scanning plaster models. Most researches evaluate the reproducibility and accuracy of the scanner by comparing the virtual models obtained from the indirect method with the plaster models. The size of tooth, width of arch etc. are usually compared by comparing the measured values of the plaster model with use of a digital caliper to the values obtained from scanned images [2–4]. But there are many factors which may cause errors in these methods, so as of recently there has been an introduction of analytical methods which employ 3-dimensional superimposition for comparison and analysis [5]. Images obtained from scanners are not only used in the general production of dental prostheses, but also as guides in implant operations and the production of implant prostheses, and more recently, in the diagnosis and planning of orthognathic surgery and orthodontic setups [6–10]. This signifies that operations that analyze the manner of occlusal contacts and the state of the occlusal plane of the complete maxillary and mandibular dentition using digital images has become commonplace.

As a result, new methods that analyze the occlusal points, the occlusal area and the occlusion have surfaced as a research topic. The T-scan and the Prescale are representative [11]. With the T-scan, the patient is instructed to carry out a series of occlusal movements such as lateral movements and anterior movements with a sensor placed in between their upper and lower arches, which is then recorded making it possible to analyze the order of occlusion according to the duration and strength of pressure applied on the tooth. The T-scan is recognized as a reproducible method in the analysis and evaluation of occlusal contacts at maximum intercuspation [12–15]. The Prescale system which was developed in Japan in that early 1990s consists of a pressure sensitive film in which a pressure between 5Mpa and 150Mpa applied to it will result in the destruction of micro capsules within the film, and a red color appears as a colorless dye mixes with a developing solution in the film. The color becomes darker as the pressure applied to it is higher, and the difference in the density of the color is analyzed. Hattori et al. [16] stated that the Prescale was meaningful as scientific data since it is possible to compare data obtained in a large scale research such as cohort studies. Suzuki et al. [17] stated that the speed and duration of the strength of occlusion applied to the Prescale sheet does not influence any changes in its color, and that the Prescale sheet does not get influenced by the moisture in the oral cavity. Also, according to the research conducted by Hidaka et al., [18] it was reported that there was a strong linear relationship between the load applied to the Type-R 50H sheet and the load deciphered from the sheet, making the Prescale it a trustworthy system in measuring the occlusal force.

Orthodontic treatment is the solution of malocclusion and the creation of functional occlusion. For 3-dimensional imagery to be generalized in orthodontic treatment, evaluation of the accuracy of the virtual occlusion obtained from the intraoral scanner is required. Research on the occlusal contact of restorations obtained from single tooth or partial arch scans are ongoing, but research on the occlusal contact of the full arch using virtual occlusion is meager and its accuracy has not been proven [19–21]. Before this study was conducted, clinically when the virtual occlusion was constructed, errors were commonly seen in certain area. Even though there was a lack of contact in the anterior portion of the maxilla and mandible, contact between the two arches was indicated. But because of the limited space when directly scanning the patient’s mouth leads to a decrease in accuracy, [22–25] a cast study which can control these conditions and increase reproducibility is in need. Therefore, the aim of this study was to evaluate the accuracy of virtual occlusions obtained by intraoral scanners using plaster dental models in vitro, by comparing virtual occlusal contact area with actual occlusal contact area obtained by prescale method.

## Methods

### Study materials

#### Study plaster models

Of the patients that visited the Orthodontics This study Department in Ewha Womans University Mokdong Hospital who were over 19 years of age, before orthodontic treatment, 24 sets of Class I models and 20sets of Class II models were selected. The criteria on model selection were as follows.

1. Models with an orthodontic base.
2. Models with Class I or Class II division 1 M occlusal relation.
3. Models without any fractured cusps or incisal edges.
4. Models of patients before any orthodontic treatment.
5. Models containing only permanent teeth, excluding the 3rd molar.
6. Models without any ankylosed teeth or missing teeth, excluding the 3rd molar.

Models with the following criteria were excluded from this study.

1. Models with an arch length discrepancy of 5 mm or over due to crowding or spacing.
2. Patients were excluded when there were additional plaster models and bite materials for Temporomandibular Joint disorder diagnosis and treatment.

Scanning commenced after surface air bubbles and other minor deformations were removed from the selected models.

### Method of research

#### Obtaining the 3D digital scan model

**Fig. 1:**
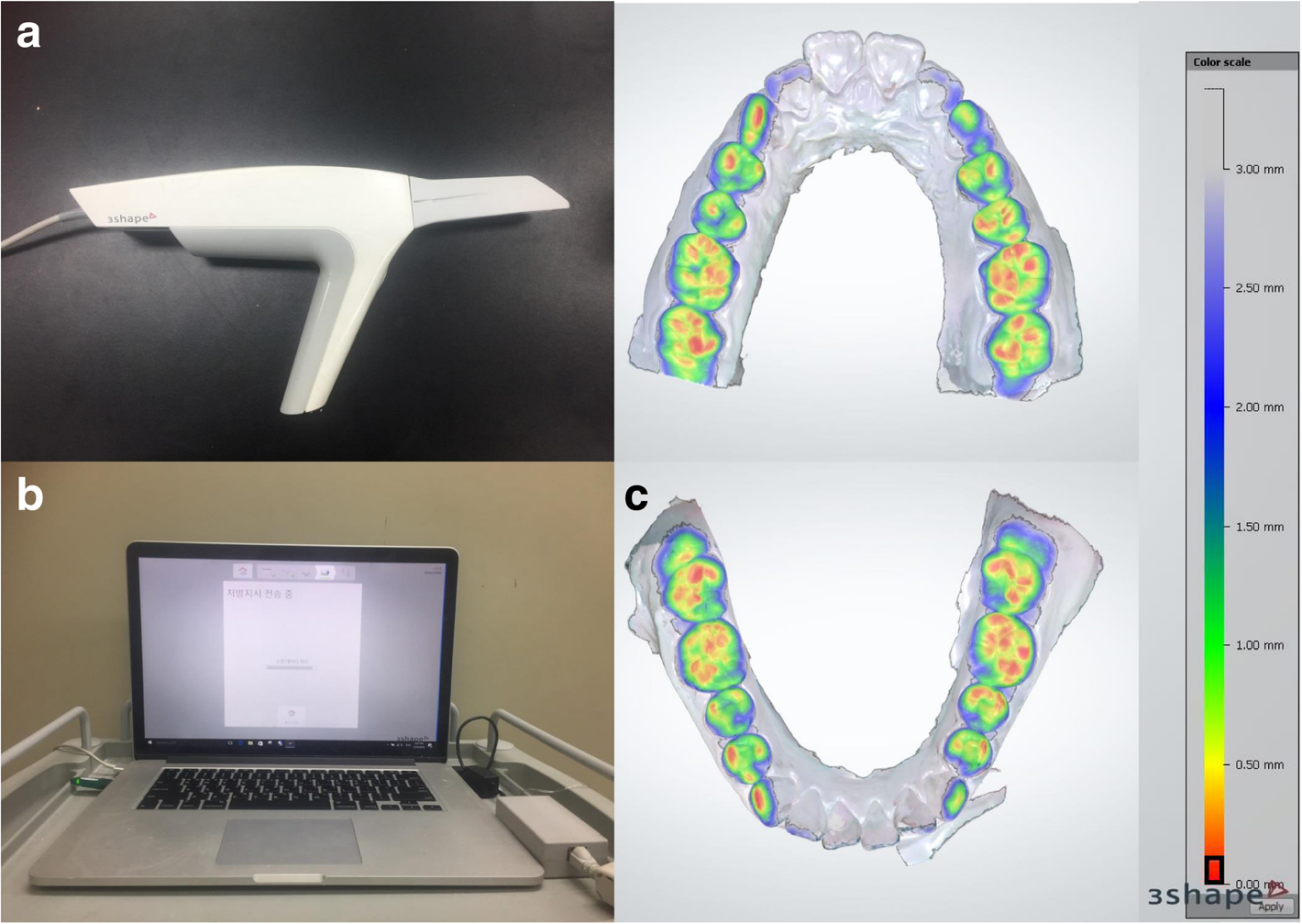
3D digital intraoral scanner(Trios®). **a** Trios® pod; handheld, lightweight pod of Trios®. **b** Scanner can be connected to personal computer, and therefore easily accessible in clinic. It displays real-time scanning process. **c** Color coded occlusal map and color scale by Orthoanalyzer® program. Color scale indicates a range of distance between maxillary and mandibular teeth. Interocclusal distance increases as the color changes from red to blue scale. The distance values are shown in millimeter

The 3D intraoral scanner used in this study was the 2nd generation Trios®(3 shape dental systems, Copenhagen, Denmark). With the Trios, scanning of the maxilla and mandible, and automatic occlusal setting is possible. By scanning the buccal side when the two arches are in occlusion, the Trios immediately reproduces the occlusive state of the models. Occlusal analysis is carried out in the form of an occlusal map which is presented in different colors depending on the distance from the surface of the tooth to the opposing tooth in the opposing arch (Fig. 1). Using this occlusal map, analysis of the occlusion and interference existing in the maxillo-mandibular complex can be carried out.

Scanning was carried out by one researcher, **HML** after thorough training on the use of the scanner. All the models were scanned in the order suggested by the manufacturer. All the models were scanned to the bucco-lingual boundary of the base, but the floor of the base was not scanned. The direction of the scans were carried out in identical fashion and based on the knowledge that inclusion of the palate influences the accuracy of the scan, the palate was not included in the scans [26, 27]. Scanning was commenced from the right 2nd molar, following the occlusive surface up to the left 2nd molar. Scanning continued onto the palatal surface of the left 2nd molar all the way to the right 2nd molar, including a portion of the occlusal surface. Finally, the buccal surface was scanned from the right 2nd molar to the left 2nd molar. After this primary scan, the final scan was completed by supplementing any insufficient portions. All maxillary and mandibular models were scanned in an identical manner.

#### Obtaining the occlusal relationship

##### Obtaining the occlusal contact area using the Prescale

The Dental Prescale system (Fuji Film Corp., Tokyo, Japan) consists of a pressure sensitive sheet in the form of the dental arch and a CCD camera which deciphers the sheets. The pressure sensitive sheets are divided into a thin R-type (98 μm thick) and a W-type (800 μm) which is covered in wax. Depending on the range of measurement, they are further divided into a 30H type and a 50H type. The 50H, R-type films were used for this study.

**Fig. 2:**
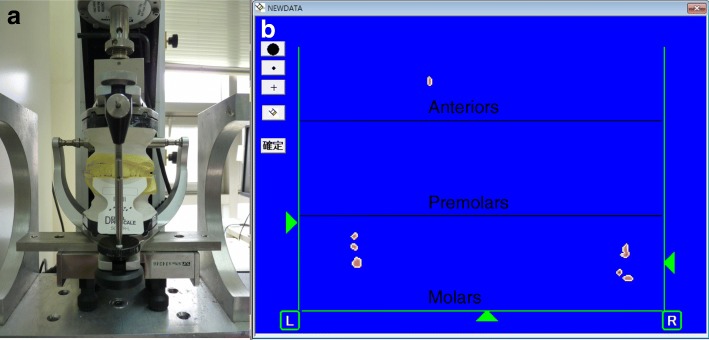
The occlusal contact area obtained by the prescale. **a** With help of the Instron, models and pressure-sensitive film are applied a compressive force of 600 N for 5 s. **b** Occlusal contact areas were shown by prescale. The area where the anteriors, premolars and molars’ position was marked on the space next to the film. The occlusal contact area and the occlusal force are automatically calculated

The position of different groups of teeth was marked on the empty spaces on the Prescale pressure sensitive sheet. At the position where the maxillary and mandibular 1st molars were occluded, a load of 600 N was applied for 5 s using the Instron. Then the occlusal strength and occlusal area indicated on the pressure sensitive paper was calculated up to a unit of 0.1mm^2^ using the CCD camera (OccluzerⓇ FPD 707, Fuji Film Corp., Tokyo, Japan) (Fig. 2).

The tooth groups were divided and compared as follows.

- Molars: 1st and 2nd molars were included. 3rd molars were excluded
- Premolars: 1st and 2nd premolars were included
- Anteriors: The Central Incisors, Lateral Incisors and Canines were included

##### Obtaining the occlusal relationship using the scanner

All maxillary and mandibular models were mounted on an articulator (KaVo PROTAR Evo 5®, Kavo Dental GmbH, Riss, Germany). A pilot study was conducted to reproduce the occlusive force exerted onto the plaster models based on the research of Yoon et al. [28] which concluded that the average strength of occlusion in Koreans with malocclusion was 439 ± 229.9 N. Based on this pilot study, a load of 600 N was decided upon for the extent of this research.

**Fig. 3:**
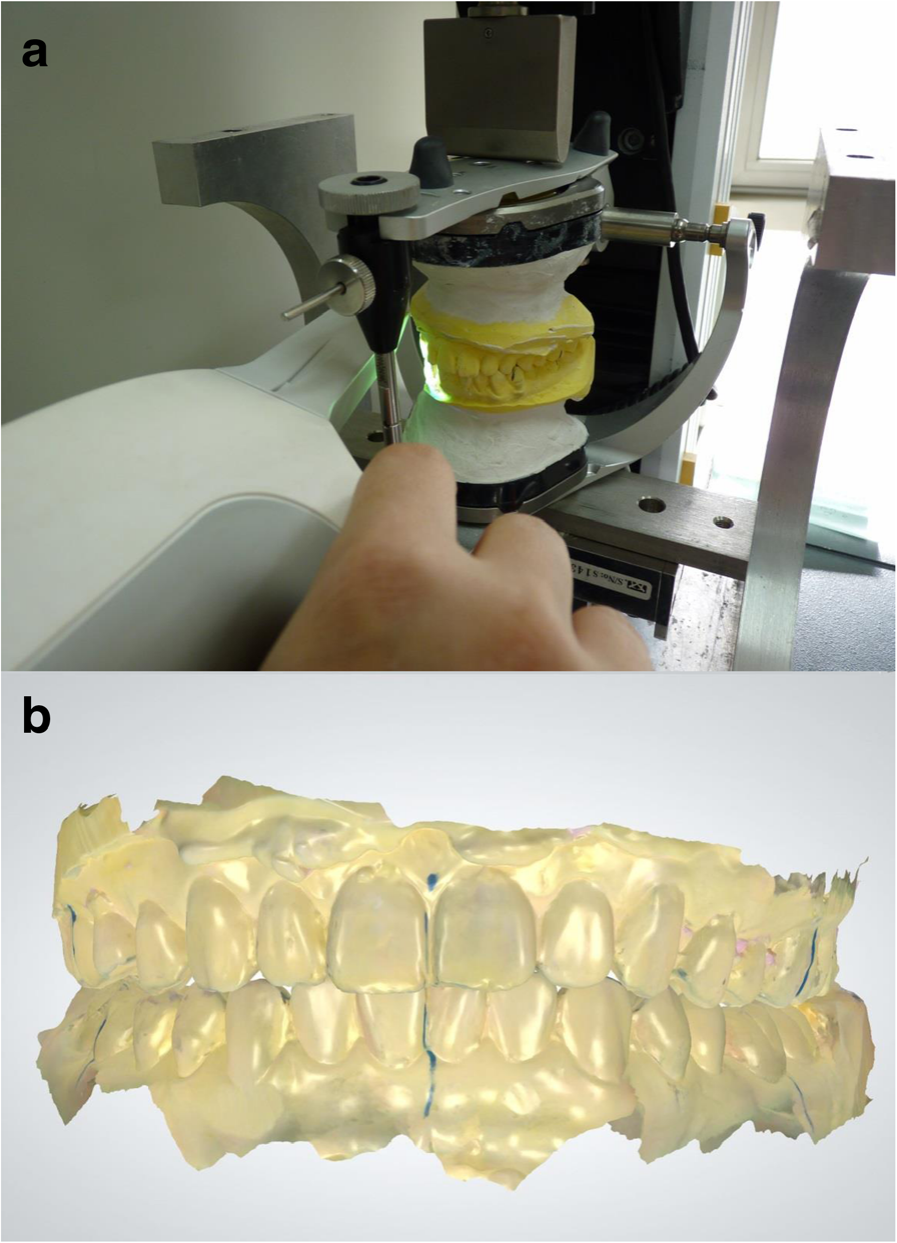
Scanning models and virtual bite registration. **a** With occluded models, the models were mounted on the articulator and compressive force(600 N) of Instron, thereafter buccal/labial facet is scanned by Trios. **b** Digital intraoral scanner(Trios®) automatically captures occlusion images and instantly register and validate patient’s bite

With the models in occlusion, the buccal surface of the models were scanned while applying a load of 600 N with the Instron(Instron, Canton, MA, USA). The direction the scans were taken were identical in all the models. Starting from the buccal surface of the right 2nd molars, the scans were continued onto the labial surface of the anterior teeth onto the left 2nd molars. After the scan was completed, a color coded occlusal map of the virtual occlusion was obtained (Fig. 3).

**Fig. 4:**
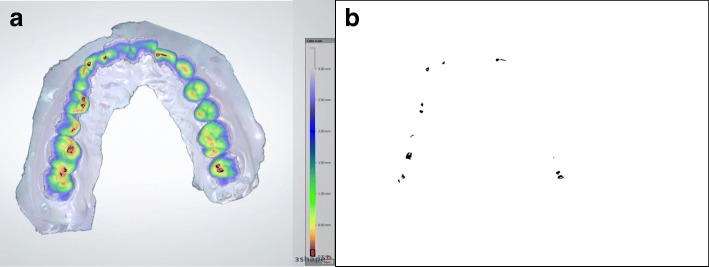
Color coded occlusal map and virtual occlusion obtained by scanning. **a** Using the color scale, the black scale corresponds to 0.0–0.098 mm of the interocclusal distance. **b** The black marking area corresponds to black scale in Fig. 4-A. Using photoshop, numbers of pixels in the black marking area were counted

Taking into consideration the 0.098 mm thickness of the Prescale sheet, the area calculated from the scan was 0.098 mm of the interocclusal distance between the maxillary and mandibular teeth. Using Photoshop (Adobe Photoshop CS3 software., Adobe Systems Inc., San Jose, USA) the number of pixels contained in the area corresponding from 0 mm to 0.098 mm on the color coded scale was calculated (Fig. 4).

#### Comparison of the occlusal contact area

The unit of the occlusal contact area obtained from the Prescale was mm^2^ and the unit of the occlusal contact area obtained from the scanner was the pixel. To compare the two methods, the relative ratio of the results obtained from each method was compared. The occlusal contact area obtained from each method was converted to a percentage(%) of the total occlusal contact area in each tooth group.

##### Statistical analysis

The collected data was run through the IBM SPSS Statistics ver. 23.0 (IBM Co., Armonk, NY, USA) for statistical analysis. The paired t-test was carried out in order to compare the occlusal contact area of the virtual occlusion resulting from the 3D intraoral scanner and the occlusal contact area resulting from the Prescale. Simple regression analysis and the Pearson correlation analysis was carried out to determine the correlation between the two groups The confidence interval was set at 95%, and the significance level was set at *p* < 0.05.

## Results

### Comparison of the occlusal contact area

**Fig. 5:**
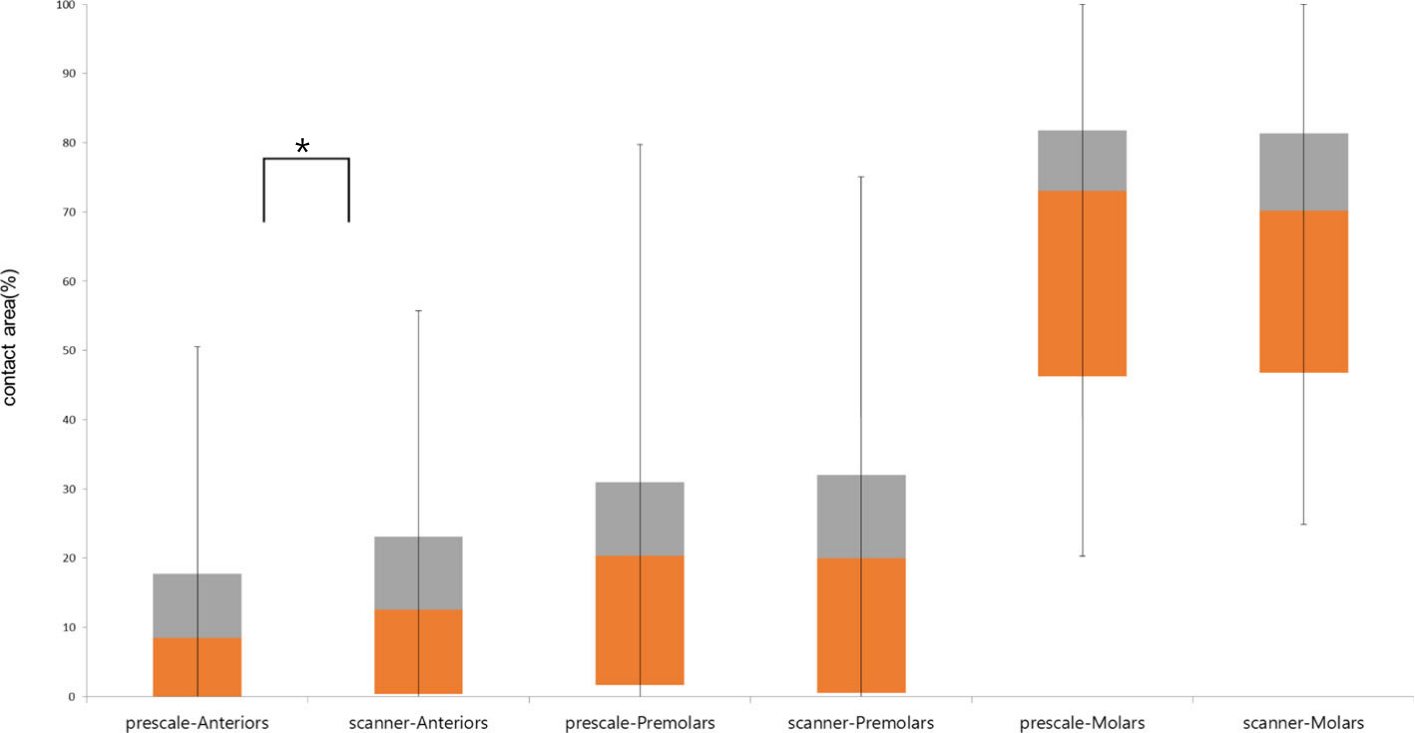
Comparison of box plots between occlusal contact area by intraoral scanner and prescale. Occlusal contact area (%) by intraoral scanner is similar in prescale for the group of Molars and Premolars. In Anteriors group, occlusal contact area by intraoral scanner is greater than prescale. The plots shows median, 25% quartile, 75% quartile and maximum-minimum range. Significance level: *; *p* < 0.05 by paired t-test

The tooth group that showed the least amount of deviation when comparing the occlusal contact area obtained from the Prescale method and the 3D intraoral scanning method was the molar portion. The ratio of the occlusal contact area obtained from the Prescale and the scanner in the molar and premolar groups were similar, and there was no statistically significant difference (*p* > 0.05). The anterior portion showed the highest amount of deviation and a statistically significant difference (*p* < 0.05). Compared to the Prescale, the occlusal contact area resulting from the scanner was overestimated. The occlusal contact area indicated in the scanning method showed overall overestimation in the anterior portion, even in the areas where there was no actual contact (Table 1) (Fig. 5).

**Table 1:** Comparison of differences between occlusal contact area calculated by intraoral scanner and prescale (prescale-intraoral scanner) (unit:%)

	Difference(SD) (prescale-scanner)	*p*-value
Molars(M)	1.59 (5.67)	0.083
Premolars(P)	1.84 (5.83)	0.053
Anteriors(A)	−3.43 (6.09)	< 0.001^*^

Difference: occlusal contact area from prescale-occlusal contact area from scanner

*SD* standard deviation

Significance level: *; *p* < 0.05 by paired t-test

### Simple regression analysis and correlation analysis

The correlation coefficients (R) of the occlusal contact area resulting from the Prescale and the intraoral scanner in all three groups showed positive correlation and were statistically significant. When comparing the values of each correlation coefficient, the molar group was the largest with a value of 0.964, that of the premolar group was 0.962 and the value of the anterior group was the smallest 0.898. This shows that there is a strong linear relation between the two variables in the molar group. In the molar group which had the highest correlation coefficient, the value of standardized regression coefficient (Β) was 0.917. The value of the coefficient of determination (R2) was 0.930 which can be interpreted in a way that it explains 93.0% of this regression model. (Tables 2 and 3).

**Table 2:** Correlation coefficients (R) and *p*-value analysis by tooth groups

Groups	R	*p*-value
Molars	0.964	< 0.001^*^
Premolars	0.962	< 0.001^*^
Anteriors	0.898	< 0.001^*^

Significance level: *; *p* < 0.05 by Pearson correlation analysis

**Table 3:** Regression of virtual occlusion by intraoral scanner on occlusion by prescale

Group	Regression coefficient		SE	*p*-value	R^2^
Molars	*B*	0.917	0.040	< 0.001^*^	0.930
	constant	3.930	2.807	0.169	

*B* standardized coefficients, *SE* standard error; R^2^, coefficient of determination

Significance level: *; *p* < 0.05 by simple regression analysis

**Fig. 6:**
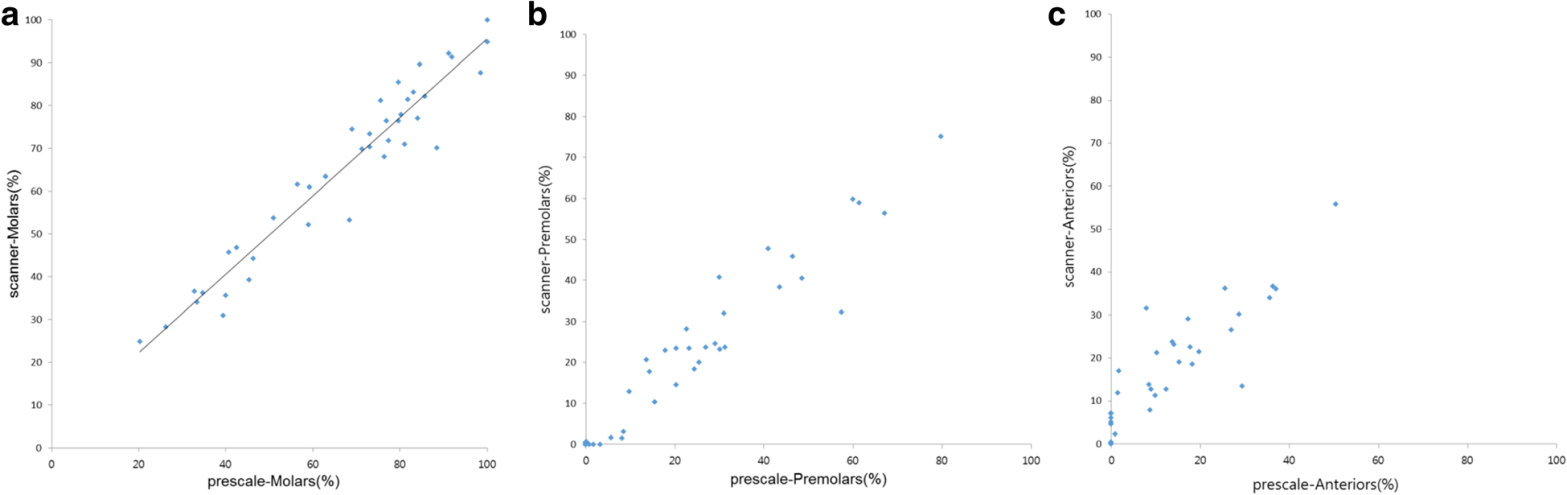
Scatterplot and regression line comparing the occlusal contact area of intraoral scanner to the occlusal contact area of prescale. **a** In Molars group, the regression analysis shows that the two variables express linear correlation and has a linear equation with a slope of 0.917. R2 is 0.930. **b** Scatterplot of occlusal contact area by intraoral scanner and by prescale in Premolars Group. **c** Scatterplot of occlusal contact area by intraoral scanner and by prescale in Anteriors Group. Groups of Premolars and Anteriors have a week linear relationship and greater dispersion

The level of dispersion of the two types of occlusal contact area and the occlusal contact area from the scanner that was estimated from the occlusal contact area from the Prescale in the molar group showed the most uniform distribution along the regression line. The values appear most scattered around the regression line in the anterior portion, with the premolar portion lying in between the molar portion and the anterior portion. As the values of the occlusal contact area gets smaller in the premolar and anterior group, they are positioned closer to the regression line (Fig. 6).

## Discussion

Currently, intraoral scanners are not only used in obtaining images of the single tooth and the dental arch, they are also utilized in the diagnosis and planning of orthognathic surgery and orthodontic setups [7, 9, 10, 29]. Using digital images obtained from scans to analyze the state of occlusal contact as well as the morphology of the occlusal surface of the complete maxillo-mandibular dentition has become more common. For intraoral digital scanners to be used more generally, it must be possible to obtain precise scan images of the full arch and dentition, and the virtual occlusion that results from those images must be able to reproduce the actual occlusion.

This study measured and compared the occlusal contact area of actual models obtained from the Prescale and the virtual occlusion obtained from scanned images. The results show that in the molar portion, there was little difference in the occlusal contact area obtained from the virtual occlusion and that obtained from the Prescale and there was no statistically significant difference. (*p* > 0.05) In the molar portion, the ratio of the occlusal contact area of the virtual occlusion to the Prescale showed a high degree of correlation, almost forming a linear line.(*R* = 0.964, B = 0.917) There was a statistically significant difference in the occlusal contact area of the virtual occlusion and the Prescale in the anterior portion, (*p* < 0.05) and compared to the occlusal contact area obtained from the Prescale, the occlusal contact area obtained from the virtual occlusion was overestimated. During the course of the research, there were a lot of cases where occlusal contact was expressed in the anterior portion of the virtual occlusion even though there was no contact in the same region according to the Prescale.

Compared to a single tooth or a portion of the arch, scanning the full arch is technically difficult and there is more chance of error when obtaining the images [30]. It has been mentioned in a number of previous studies that the magnitude of these errors are clinically acceptable [5, 30, 31].

The results of superimposition carried out by most of the previous studies in the literature aimed at evaluating the accuracy of the intraoral scanner show a lower degree of accuracy in the distal portion of the molars and the incisal surface of the anterior teeth [5, 31, 32]. These errors occur due to the presence of sharp curves in the distal portions of the molars and the incisal portions of the anterior teeth which cause diffraction of light resulting in errors when obtaining images [27, 33]. In other words, precise scan images of complex structures with the presence of undercuts are difficult to obtain. Even in this study, precise scan images could not have been obtained in the anterior portion due to the diverse morphology and tooth crowding present during the individual scanning of the maxillary and mandibular models.

The algorithm involved in the manner of registration of 3-dimensional images of the intraoral scanner could have been the cause of the results in the anterior portion. The scanner used in this study employs the best fit algorithm. There have been many studies on the best fit algorithm [34, 35]. The most representative problem with this method is that the errors that occur in the beginning continue to add up as the scanning process continues. Solaberrieta et al. [36] recommended that in order to reduce the errors in the best fit algorithm, when employing the buccal scan method to produce the virtual occlusion, instead of scanning the full arch, to only obtain three precise scan images (images of the bilateral molar area and the frontal anterior image) of a width of 24 mm and a height of 5 mm. They stated that if these three images can be obtained, a virtual model occlusion that is closest to the actual model occlusion was possible. The important point was that the distance among the sections should be as large as possible, and that precise images of the left and right molars were imperative. In reality, with the models in maximum intercuspation, when the buccal surface is scanned from the molar to the anterior portion, even before the full arch is scanned, the program completes the occlusion. Due to the best fit algorithm, as scanning progressed from the molar portion to the anterior portion, it seems that the errors added up as the scanning process continued to present itself with overestimation in the anterior portion in this study.

Intraoral scanner used in this study employs ultrafast optical sectioning technology based on confocal microscopy, taking more than 3000 2D images per second and then combining them into 3D. In the process of creating the virtual occlusion, the scanner determines the relative position of the maxilla and the mandible through the process of scanning, and depends on the images of the teeth that are already registered, as well as the images of the surface of the maxillary and mandibular teeth in intercuspation. But these images do not include any information on the direction, position or angle in a 3-dimensional space [21, 34]. Due to the lack of 3-dimensional information, it is difficult to position the scanned images of the maxilla or the mandible when there is a partial or complete edentulous area. And detailed registration of superimposed images of two scans is also difficult [34]. As it is shown in the results of this study, when the virtual occlusion is formed from the registration of the two images, the anterior portion is shown to contact more closely than in reality. Also, the maxillo-mandibular labial images that are superimposed as a result of the diverse overbite and overjet in the anterior labial portion may not have supplied adequate information in the registration of the maxilla and mandible, which could be the cause of another form of error. In reality, to solve these problems, methods for precise registration are being suggested and effort in developing such software is underway [37, 38]. Research uses the virtual articulator to construct the virtual occlusion from scanned images [37, 39]. Delong et al. [37] demonstrated the possibility of reproducing the functions of the virtual articulator and actually obtaining acceptable results by comparing its accuracy under various conditions, and creating the necessary software to analyze the occlusion from virtual models.

The Prescale which was used as a control group in this study makes it possible to analyze the occlusal contact area of the full arch quantitatively, and is known as a trustworthy system in measuring the occlusive force. It can be with better use as it enables to compare scientific data when large data is collected [15, 17]. The Prescale is a good means to quantitatively evaluate the occlusive force, but it has its limitations in the form of the deformation of its sensors and its thickness. The thickness of the film may interrupt movement onto intercuspation and it has been reported that there is a limitation as a result of the distortion of the pressure sensor [15].

But currently, of the methods of measuring the occlusive force, since there is no golden standard that can represent the occlusal contact points, the Prescale which is a scientific method that can display the occlusive force was used in this study.

Being conducted with plaster models, the results of this study could be different from clinical tests. Errors of the Prescale resulting from the non-uniform pressure upon closing or the position of the head, by applying uniform pressure to the models with the Instron were avoided but it was not possible to express the maximum intercuspation through the opening and closing of the mandible in an actual oral cavity. However it can be a good milestone to develop the software for 3d intraoral scanner. And it can examine the difference between virtual and real occlusal by scanning system as it reduces the errors from intraoral scanned images and imaging skills.

## Conclusion

This study evaluated the accuracy of the jaw relation record through the buccal scan method in a state of occlusion by comparison of the occlusal contact area obtained by the Prescale and a 3D intraoral scanner, and whether it could be used clinically. The tooth groups were divided into a molar portion, a premolar portion, and an anterior portion. The occlusal contact area obtained from the Prescale and the 3D intraoral scanner was measured, ultimately comparing the virtual occlusion and the actual occlusion.

In the molar portion and the premolar portion, there was no statistically significant difference between the occlusal contact area obtained from the Presacle method and the scanning method (*p* > 0.05). There was a statistically significant difference between the two methods in the anterior portion where overestimation was observed. (*p* < 0.05).

There was no statistically significant difference between the actual occlusion and the virtual occlusion using 3D scan images in the molar portion, but there was statistically significant difference between the two methods in the anterior portion, which presented with overestimation of the occlusal contact area obtained from the scanning method. The virtual occlusion using scanned images can be used as a reference in the diagnosis and modification of the occlusal contact. But there are limitations to the scanning method, therefore this should be taken into consideration when applying it in the clinical environment. Software and scanning techniques that can more precisely reproduce the patient’s jaw relation must be developed.

## Abbreviation

3D: 3 Dimensional
Cl: Class
SD: Standard deviation
SE: Standard error
